# Effects of *Launaea taraxacifolia* and resveratrol on milk yield and serum prolactin and oxytocin levels: a lactogenic study

**DOI:** 10.1080/23144599.2019.1694307

**Published:** 2019-12-17

**Authors:** Na’imatu A. Sani, Mohammed U. Kawu, Ibrahim G. Bako

**Affiliations:** a Department of Veterinary Physiology, Faculty of Veterinary Medicine, Ahmadu Bello University, Zaria, Nigeria; b Department of Human Physiology, Faculty of Basic Medical Sciences, College of Medicine, Ahmadu Bello University, Zaria, Nigeria

**Keywords:** Antioxidant, herbal galactogogue, lactogenesis, oxytocin, prolactin

## Abstract

Inadequacy of milk supply to meet the increasing human population coupled with a decreasing livestock population has necessitated the need for a potent galactogoue. The aim of this study was to compare the lactogenic effects of *Launaea taraxacifolia* (PLT) and resveratrol in Wistar rats. After parturition, 25 primiparous female Wistar rats were randomly allocated into five groups of 5 dams each. Dams in groups I, II, III, IV and V were administered distilled water (DW: 2 ml/kg), metochlopromide (MET: 15 mg/kg), resveratrol (RES: 5 mg/kg), *n*-hexane leaf fraction of *L. taraxacifolia* (PLT: 333 mg/kg) and the combination of RES + PLT (CO: 5 mg + 333 mg/kg); respectively, for 12 days. Pup weight gain was used to quantify milk yield. Serum was harvested from the dams and assayed for prolactin and oxytocin. The PLT and CO groups had significantly higher (*p* < 0.05) milk yield than DW group. Serum concentration of prolactin was significantly higher (*p* < 0.05) in the PLT group, while the combination group had the highest (*p* < 0.05) concentration of oxytocin compared to DW group. In conclusion, *L. taraxacifolia* and resveratrol exhibited galactopoietic potentials individually by stimulating hyperprolactinaemia, while their combination increased milk production by increasing serum oxytocin activity.

## 1. Introduction

Gross deficiency of protein intake both in quantity and quality is a potent food security problem, particularly in developing countries. The insufficiency of cow's milk supply to increasing human population in developing countries has resulted in a high number of non-dairy milk substitutes for cow'smilk. However, cow milk has higher protein content and quality than the non-dairy milk beverages [1]. Malnutrition, low productivity and high incidence of preventable diseases have been attributed to low protein intake [2]. Milk is a complete source of nutrients, particularly protein necessary for growth, development and maintenance of healthy living. It also facilitates recovery from malnutrition [3]. Bhutta et al. [4] reported that annual death of over 823,000 children and 20,000 mothers could be prevented by strict implementation of universal breastfeeding, and this could contribute to global economic benefits to the tune of US$300 billion in savings.

Milk production (lactogenesis) is a complex neuro-endocrine activity that involves interaction of a number of physical and emotional factors along with the action of multiple hormones, mainly prolactin [5] and oxytocin. Prolactin and oxytocin are the most important hormones necessary for mammary milk production and milk delivery to the young, respectively [6]. The secretion of these hormones is stimulated by the suckling action of the young at the teat/nipple [7]. Dopamine agonists and antagonists control milk production by regulating prolactin synthesis and secretion through interaction with the hypothalamus and anterior pituitary gland [8,9]. Lactogenesis and galactopoiesis are stressful processes for lactating animals. Since hormonal control of lactation is affected by stress [5], control of stress in lactating animals could be an important way to enhance lactation. Therefore, phyto-pharmacological research on natural products with significant antioxidant activity could serve as an important tool for the discovery of new active compounds with novel structure and potential to serve as natural lead compounds for development of new galactogogues. Galactogogues are foods, herbal medicines or pharmaceutical drugs used to induce or enhance milk secretion [10].

Although some studies have reported the ability of some medicinal plants to increase milk production by stimulating prolactin release [11–14]; we have reported for the first time, the comparative effects of a medicinal plant and resveratrol (a potent antioxidant) on milk production, serum prolactin and oxytocin concentrations.

## 2. Materials and methods

### 2.1. Plant collection, identification and extraction

Fresh leaves of *Launaea taraxacifolia* were obtained in Zaria, Kaduna State of Nigeria. The leaves, flowers and seeds of the plant were sent to the Herbarium Unit of the Department of Botany, Faculty of Life Sciences, Ahmadu Bello University, (A.B.U.), Zaria, Nigeria for identification and voucher number of 648 was deposited. The leaves were dried in open air in the laboratory to a constant weight. The dried leaves were ground to fine powder with mortar and pestle and 1.19 kg of the pulverized leaf was extracted with 5 litres of absolute methanol using percolation. The filtrate was concentrated with rotating evaporator.

### 2.2. Fractionation of crude methanol extract

The crude methanol extract was serially fractionated with *n*-hexane and ethyl acetate using a separating funnel. Each partition process was repeated twice using equal volume of each solvent, and similar fractions were pooled together and concentrated using rotating evaporator.

### 2.3. Phytochemical screening

Thin layer chromatographic plate (Merck, Germany) was used to develop chromatograms for the identification of phytoconstituents in the fraction. The plate was developed in hexane:ethyl acetate in a ratio of 9:1 and sprayed with ferric chloride (phenols), aluminium chloride (flavonoids), dragendoff (alkaloids), Liebermann-Burchard (steroids and triterpines) and Bontragers (anthraquinones) reagents. The chromatograms were visualized under day light, ultraviolet light and then heated at 100°C.

### 2.4. Experimental animals

Thirty-five adult male and nulliparous female rats comprising 30 females and 5 males, weighing about 120–190 g were obtained from the Animal House of the Department of Veterinary Physiology, Faculty of Veterinary Medicine, A.B.U., Zaria. Ethical approval for the study was obtained from the Ahmadu Bello University Committee on Animal Use and Care (ABUCAUC) and approval number ABUCAUC/2018/053 was obtained.

### 2.5. Breeding of experimental rats

Thirty rats were divided into 5 groups of 6 rats per group. Each group containing 5 female and 1 male rat. Whitten effect was employed to synchronize the oestrous of the female rats in each group and subsequently, mating of the female rats. The animals were then observed for onset of pregnancy and parturition.

### 2.6. Experimental grouping

Few days prior to parturition, separate cages were provided for each dam and her pups. Immediately after parturition, body weight of each pup was taken (W_0_) and the 25 dams were randomly divided into 5 groups of 5 dams each. The number of pups was corrected to 5 pups/dam. Dams in groups I, II, III, IV and V were administered distilled water (DW: 2 ml/kg), metochlopramide (MET: 15 mg/kg), resveratrol (RES: 5 mg/kg), *L. taraxacifolia* fraction (PLT: 333 mg/kg) and combination of resveratrol and *L. taraxacifolia* (CO: 5 + 333 mg/kg), respectively. Milk yield was determined as described by Cai et al. [12], with slight modification for this study. The dams were treated daily at 19:00 h for 12 days, starting from day 2 to 14 of lactation. Milk yield determined as weight gain of pups was recorded daily (18 h after gavage), using an electronic balance accurate to 0.01 g using ATOM – A 110C weighing balance.

### 2.7. Measurement of body weight of pups

The pups were individually weighed three times daily. At 8.00 h, the first weight (W_1_) was taken and the pups subsequently, separated from the dams for 4 h. By 12.00 h, they were weighed again (W_2_) and then taken back to their dams to suckle for 1 h. Finally, at 13.00 h, they were weighed again (W_3_) and then allowed to stay with their respective dams for the rest of the day.

### 2.8. Determination of milk yield

Milk yield 18 h after the administration of the extract was estimated as W_3_–W_2_.

Daily pup weight was indicated as W_1_, which represents daily weight gain per pup.

Percentage weight growth rate (WGR), which indicates the growth of pups in relation to their birth weight was calculated using the following formula:

$$\%WGR=\;\;\left(W_{1}-W_{0}\right)/W_{0}\;\;\;\left[12\right]$$

where W_0_ = birth weight of pups.

### 2.9. Determination of serum levels of lactogenic hormones

On the 14th day of experiment, three dams from each group were euthanized using ketamine (300 mg/kg) followed by cervical dislocation. Then 3 ml of blood was collected from each dam into plain sample bottles. The blood was left in the laboratory bench for 30 min in slanting position to coagulate. The blood samples were centrifuged at 3000 *g* for 10 min. The serum was harvested into appropriately labelled plain sample bottles.

#### 2.9.1. Prolactin

Serum levels of prolactin were measured with rat-specific prolactin kit according to manufacturer’s instructions (FineTest, Wuhan Fine Biotech Co., Ltd., China). The principle of the test was based on competitive-ELISA detection method. After carrying out the assay, the concentration of prolactin in the samples was then determined by comparing optical density of the samples to the standard curve. The assay has a sensitivity of <7.8 pg/ml with intra-assay and inter-assay coefficients = CV <8% and <10%, respectively where CV (%) = SD/mean × 100.

#### 2.9.2. Oxytocin

Similarly, serum levels of oxytocin were measured with rat-specific oxytocin kit according to manufacturer’s instructions (FineTest, Wuhan Fine Biotech Co., Ltd., China) as described for prolactin.

### 2.10. Statistical analysis

Data obtained were expressed as mean ± SEM. Two-way and repeated-measures analysis of variance (ANOVA) was used, followed by Tukey post-hoc test for multiple comparisons of the groups. The statistical package, GraphPad Prism version 5.1 was used for analysis. Values of *p* < 0.05 were considered significant.

## 3. Results

### 3.1. Phytochemical screening

The specific TLC chromatogram was positive for only Liebermann-Burchard spray. Seven spots were seen under day light. Upon spraying with Liebermann-Burchard and heating, four of the spots turned green while the remaining three turned purple, indicating the presence of steroids and triterpenes.

### 3.2. Milk yield

**Figure 1.**
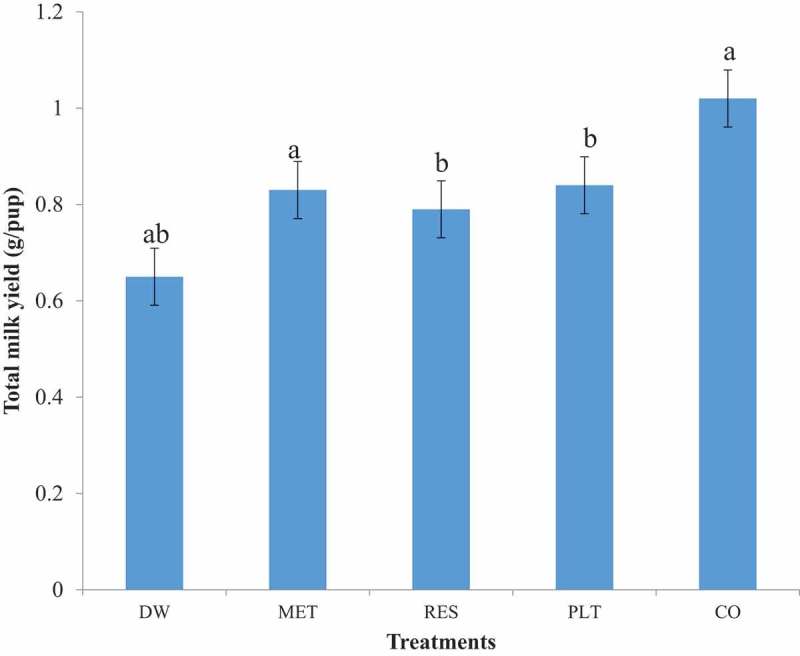
Total milk yield of dams treated with *n*-hexane fraction of the leaf extract of *Launaea taraxacifolia*, resveratrol and their combination. Bars with different superscripts are significantly different (*p* < 0.05).

The total milk yield in dams treated with CO and PLT was significantly higher (*p* < 0.05) than the DW control group (Figure 1). Similarly, the total milk yield in CO-treated group was significantly higher (*p* < 0.05) than in MET and RES groups. The total milk yield in the CO-treated group was higher than that of PLT group, though the difference was not statistically significant (*p* > 0.05). The total milk yield in PLT-treated group was higher than in RES group, though not statistically significantly, their combination (CO) resulted in the highest milk yield when compared with PLT and RES, respectively (*p* < 0.05).

### 3.3. Daily weight gain of pups

**Figure 2.**
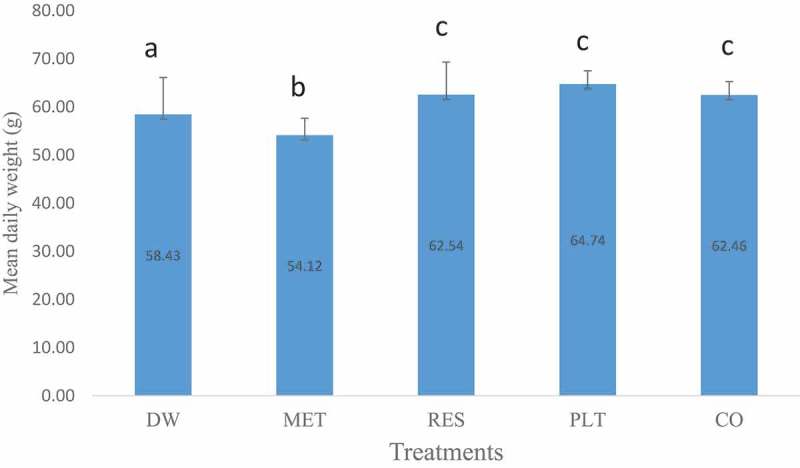
Mean (±SEM) daily weight gain of pups from dams treated with *n*-hexane fraction of leaf extract of *L. taraxacifolia*, resveratrol and their combination in lactating Wistar rats. Bars with different alphabets are significantly different (*p* < 0.05).

The mean daily weight gain of pups treated with fraction of *n*-hexane leaf extract of *L. taraxacifolia* is shown in Figure 2. The weight gain of pups was significantly (*p* < 0.05) higher in PLT when compared with DW- and MET-treated groups. Similarly, pups in RES and CO groups had significantly (*p* < 0.05) higher body weight than the MET group. However, pups in CO-treated group had lower weight gain than pups in PLT and RES groups.

### 3.4. Weight growth rate of pups

**Figure 3.**
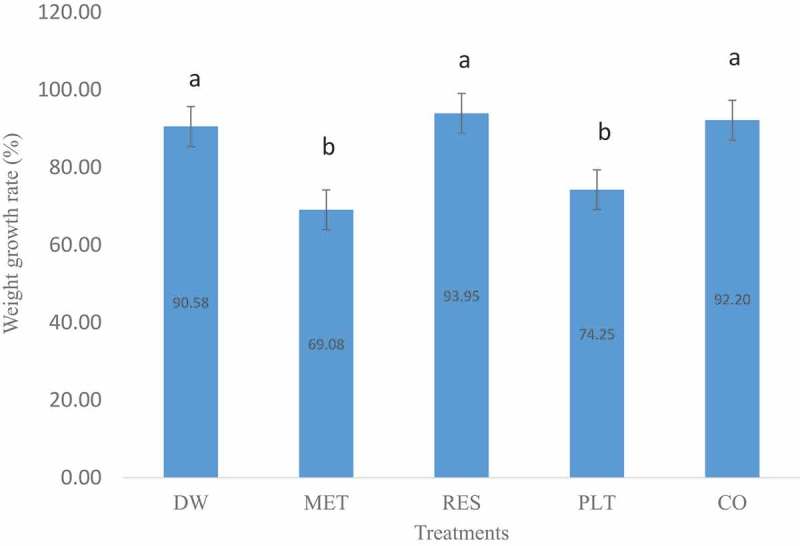
Weight growth rate of pups treated with *n*-hexane fraction of the leaf extract of *L. taraxacifolia*, resveratrol and their combination in lactating Wistar rats. Bars with different alphabets indicate statistically significantly different (*p* < 0.05).

The weight growth rate (%GR) of pups was significantly (*p* < 0.05) higher in RES and CO groups compared to MET-treated group (Figure 3). Furthermore, the %GR of RES-treated group was significantly (*p* < 0.05) higher than that of PLT-treated group. Also, the %GR of pups in RES-treated group was higher than CO group, though the difference was not statistically significant (*p* > 0.05).

### 3.5. Serum prolactin concentration

Serum concentration of prolactin was significantly higher (*p* < 0.05) in PLT- and RES-treated groups when compared to CO group (Table 1). Meanwhile, DW group exhibited lowest concentration of prolactin.

**Table 1.** Serum concentration of prolactin.

Groups	Mean ± SEM serum concentration of prolactin (ng/ml)
Distilled water	17.38 ± 0.58^a^
Metochlopramide	17.78 ± 0.46^a^
Resveratrol	51.39 ± 6.06^b^
*Launaea taraxacifolia*	60.05 ± 1.23^b^
Resveratrol and *Launaea taraxacifolia*	22.41 ± 1.45^a^

Serum concentration of prolactin in lactating Wistar rats treated with *n*-hexane fraction of *L. taraxacifolia*, resveratrol and their combination. Superscript with different alphabets are significantly different (*p* < 0.05).

### 3.6. Serum oxytocin concentration

The CO-treated group had significantly higher (*p* < 0.05) serum concentration of oxytocin compared to the other groups (Table 2). The concentration of oxytocin was also significantly higher (*p* < 0.05) in PLT when compared to the RES group. Furthermore, serum oxytocin concentration was significantly higher (*p* < 0.05) in PLT- and CO-treated groups when compared to the DW group.

**Table 2.** Serum concentration of oxytocin.

Groups	Mean ± SEM serum concentration of oxytocin (ng/ml)
Distilled water	35.69 ± 1.82^a^
Metochlopramide	53.44 ± 2.95^ab^
Resveratrol	74.44 ± 0.37^a^
*Launaea taraxacifolia*	112.6 ± 4.85^b^
Resveratrol and *Launaea taraxacifolia*	234.9 ± 8.106^c^

Serum concentration of oxytocin in lactating Wistar rats treated with *n*-hexane fraction of leaf extract of *L. taraxacifolia*, resveratrol and their combination. Superscript with different alphabets are significantly different (*p* < 0.05).

## 4. Discussion

Increased dairy production has the potential to enhance livelihood and reduce poverty in less developed countries. However, relatively low milk yield coupled with an increase in per capital income and population has resulted in milk supply shortage [15]. Increased production of milk and milk-related products could be beneficial to the well-being and economic development of these countries.

The increased milk yield seen in the PLT, RES and CO groups could be due to stimulatory effects of *L. taraxacifolia* and resveratrol on secretory activities of mammary lactocytes. Mahmood et al. [16], reported that enhanced milk production in lactating rats was attributed to the stimulatory effect of *Musa x paradisiaca* extract on cellular proliferation in the mammary gland. Plants with galactopoietic activities have been reported to affect mammary gland cellular proliferation and activity with resultant increase in milk production [17–19]. In this study, administration of PLT, RES and CO enhanced milk production in the treated rats.

The higher daily weight gain of pups seen in PLT, RES and CO, may be explained by two factors. It may be as a result of higher milk secretion of dams in these groups which led to greater quantity of milk supply to the pups for body growth. Or, it could be as a result of enhanced nutritional effects of the extract and resveratrol on the milk components. Lompo-Ouedraogo et al. [13] opined that increased pup weight was due to improvement in the nutritional value of the dam’s milk. Thus, we also hypothesize that the daily weight gain of pups observed in this study could be directly proportional to the volume and possibly, enhanced nutritional value of the milk produced by the lactating dams. Since milk consumed by the pups is for growth and maintenance, it can be inferred that the dams of PLT- and RES-treated groups produced higher amount of milk and their pups consumed more milk with resultant higher pup weight gain seen in these groups.

The highest milk yield observed in the CO-treated group may be due to the synergistic effect of the bioactive metabolites in the PLT and RES. Feed supplementation was used to improve performance of lactating animals during early lactation [20]. Therefore, oral administration of leaf extract of PLT to the lactating rats may have supplemented these animals, which provided them with sufficient nutrients for body maintenance and growth to cope with increased demand for nutrients associated with lactation. This observation may lend credence to the significantly higher percentage growth rate recorded in PLT-treated group. Percentage growth is an index used to evaluate the potency of treatment in lactogenic studies. The higher weight gain and percentage growth rate seen in RES group compared to PLT and CO groups, despite higher milk yield in the latter groups, may imply that RES enhanced utilization of milk by the pups. Milk is the only source of nutrient to these pups and its yield was lower in RES group yet the pups in this group had higher body weight gain. Resveratrol modifies microfauna of the gastrointestinal tract with resultant higher growth rate of the animal [21]. It blocks the production of glutathione disulphide and inhibits cellular damage produced by free radicals [22]. In addition to inhibition of production of free radicals, resveratrol protects DNA against oxidative stress by scavenging free radicals already generated in the body [23].

Furthermore, the higher serum prolactin (Prl) concentration recorded in PLT-treated group may underpin the mechanism of action of enhanced secretory activity of mammary glandular alveoli with resultant increased milk secretion/yield in this group. Prl is a physiological relay sensor which responds to demands for milk production and redirect nutrients away from the adipose tissues to the mammary glands for optimum milk production [24]. It is also important for mammary secretory activation that occurs at parturition and initiates the secretion of milk into lobulo-alveoli units. Since inadequate diet and or metabolic pathologies, such as, diabetes mellitus, adversely affect secretory differentiation and activities of the mammary gland with consequent poor lactational performance [25], PLT could be used to ensure adequate and sustained lactational performance in such disease conditions. Although resveratrol also increased serum concentration of Prl, the effect of CO on serum concentration of prolactin was rather antagonistic, resulting in lower concentration of Prl. Some herbal galactogogues have been shown to increase milk secretion by increasing the Prl activity [11–13]. It is worthy of note that resveratrol-treated group showed a dramatic increase in the serum prolactin concentration when administered alone. This could be due to the effect of resveratrol as a supplement that improves the general well-being of animals when taken as a single dose or for a certain period of time [26].

While Prl regulates the expression of target genes, and stimulates lipid synthesis and exocytosis through the JAK2/STAT5 pathways for sustained lactogenesis; oxytocin (OT) is released in response to the suckling reflex of the young and stimulates the contraction of myoepithelial cells with resultant milk letdown [9]. Thus, Prl promotes lactogenesis, while OT triggers milk delivery to the young. The highest serum concentration of OT recorded in the CO suggests a synergistic effect between PLT and RES in stimulating milk letdown. Augustine et al. [27] observed that Prl up-regulates the activities of OT neurons and maintains the concentrations of OT in lactating rats. In contrast, Prl inhibits pulsatile release of OT in virgin rats by causing sustained hyperpolarization of OT neurons, thus inhibiting their activity [28]. Thus, the change from Prl-induced inhibition of OT in virgin rats to Prl-facilitated release of OT during lactation occurs in favour of the young. The higher plasma concentration of OT seen in PLT and RES groups compared to the DW group suggests that *L. taraxacifolia* and resveratrol stimulates pulsatile release of OT from the posterior pituitary. Since PLT and RES caused increased serum OT concentration, while their combination showed a synergistic effect on serum OT concentration; both supplements can be said to have both lactogenic and galactopoietic effects. They perhaps act synergistically to increase OT synthesis and secretion thereby stimulating milk ejection reflex in the mammary gland. Resveratrol may have achieved this effect via its amelioration of oxidative stress. Stress affects the paraventricular and supraoptic nuclei of the hypothalamus and inhibits pulsatile release of OT and consequently decrease milk ejection [29].

Good galactopoietic effects of hexane fraction of *L. taraxacifolia* could be due to the presence of steroids and triterpenes (polyphenols) detected from the preliminary phytochemical screening of the fraction. Polyphenols increase milk yield of lactating animals by their antioxidant effects [5].

## 5. Conclusion

*Launaea taraxacifolia* and resveratrol independently enhanced milk production in lactating rats by stimulating hyperprolactinaemia. The combination of *L. taraxacifolia* and resveratrol, however, increased milk production through increased oxytocin release which acts on myoepithelial cells of the mammary glands to stimulate milk ejection reflex.
